# Biventricular Noncompaction Cardiomyopathy in an Adult with Unique Facial Dysmorphisms: Case Report and Brief Review

**DOI:** 10.1155/2015/831341

**Published:** 2015-07-21

**Authors:** Gaurav Rao, James Tauras

**Affiliations:** ^1^Division of Internal Medicine, Montefiore Medical Center, Albert Einstein College of Medicine, Bronx, NY 10467, USA; ^2^Division of Cardiology, Weiler Hospital, Albert Einstein College of Medicine, Bronx, NY 10461, USA

## Abstract

Left ventricular noncompaction (LVNC) is a rare cardiomyopathy that is believed it to arise from an arrest in embryonic endomyocardial development. More recent studies suggest that it can be acquired later on in life sporadically. It may be accompanied by life-threatening complications, which are most commonly heart failure, arrhythmias, and thromboembolic events. We report a case of biventricular noncompaction cardiomyopathy in a 36-year-old man presenting for the first time with clinical heart failure as well as atrial arrhythmia. Transthoracic echocardiography (TTE) revealed LVNC with depressed ejection fraction (EF). Cardiac magnetic resonance imaging (MRI) further revealed a left atrial appendage thrombus as well as right ventricular noncompaction involvement. His physical exam was unique for a characteristic facial dysmorphisms pattern and developmental delays reminiscent of the earliest descriptions of LVNC in the pediatric population and it was rarely described in adult patients. This unique presentation underscores the importance of a better understanding of the genetics and natural course of LVNC. This will help us to elucidate the uncertainty surrounding its clinical management, discussed in a brief review of the literature following the case.

## 1. Introduction

Left ventricular noncompaction (LVNC) is a rare cardiomyopathy that is thought to arise during embryogenesis secondary to arrested myocardial development. This results in a thickened myocardium bilayer comprised of noncompacted myocardium, characterized by prominent deep intertrabecular recesses [1] and a thin compacted layer of myocardium [2]. Its etiology is genetic or sporadic. The most common clinical manifestations are heart failure, ventricular arrhythmias, and thromboembolic events [3]. As a younger cardiomyopathy, its natural history is still being understood with optimal management yet to be determined. We present a unique case of biventricular noncompaction and its complications in an adult with unique facial dysmorphisms resulting in multiple management implications.

## 2. Case Presentation

A 36-year-old man with a three-month history of intermittent lower leg extremity edema managed with furosemide by his primary care doctor presented with worsening lower leg edema, dyspnea on exertion with decreased exercise tolerance. He had no other significant medical history apart from history of cognitive, speech, and hearing impairment since an early age. He was a nonsmoker with occasional alcohol use and no recent foreign travel. There was no family history of cardiomyopathy.

In the emergency department, electrocardiogram revealed atrial flutter with 2 : 1 conduction with a heart rate upwards of 130 beats per minute. Physical exam was significant for facial dysmorphisms with a prominent forehead, micrognathia, high arching palate, and low set ears. His jugular venous pressure (JVP) was elevated with systolic ejection murmur 3/6 heard best at apex along with bibasilar crackles on lung auscultation. Lower extremity exam was significant for pitting edema to the knees.

**Figure 1:**
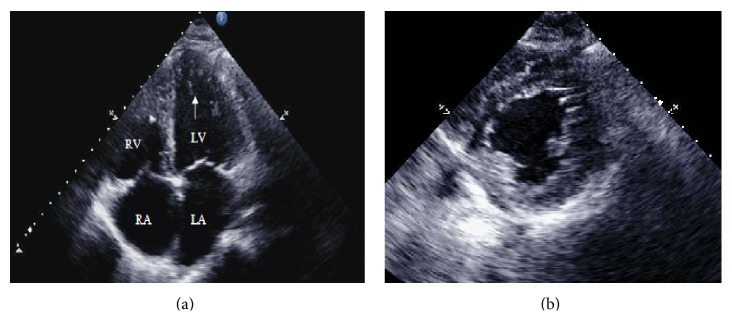
Echocardiogram of the heart. (a) Apical four-chamber view of the heart with marked trabeculations of the apex (white arrow). (b) Parasternal short axis view at the level of the papillary muscles with prominent LV inferolateral trabeculations. RA: right atrium, LA: left atrium, RV: right ventricle, and LV: left ventricle.

**Figure 2:**
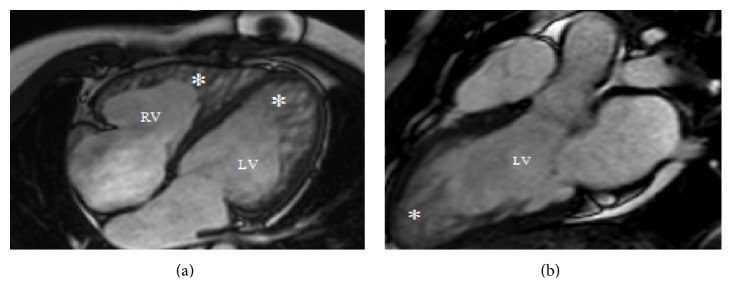
Cardiac MRI of the heart. (a) Cardiac MRI showing extensive biventricular trabeculations with (b) sagittal view demonstrating deep recesses in the left ventricle cavity (see asterisk).

Laboratory data was significant for newly elevated creatinine of 1.8 mg/dL and pro-BNP of 10,500 pg/mL. The rest of laboratory tests were normal. X-ray showed cardiomegaly and vascular congestion. Transthoracic echocardiography (TTE) revealed biatrial dilatation, severe biventricular failure with LV ejection fraction (EF) of 15%, and prominent trabeculations in the left ventricle (Figure 1). Cardiac magnetic resonance imaging (MRI) showed similar findings, but it also demonstrated biventricular, extensive myocardial trabeculations compatible with noncompaction cardiomyopathy (Figure 2).

He was begun on nitroglycerin and furosemide with pulmonary artery catheter placement and transferred to the cardiac care unit. Escalating doses of metoprolol were used for rate control. Heparin infusion was also initiated for atrial fibrillation stroke risk. Transesophageal echocardiography (TEE) was done prior to a planned cardioversion the following day, which showed a left atrial appendage thrombus.

Over the following week, our patient was aggressively diuresed and intravenous diuretics were down titrated prior to transferring out of the unit. He was commenced on beta-blocker, ACE inhibitor, and long-term anticoagulation for his thrombus. He spontaneously converted to sinus rhythm. Given the improvement in patient's EF to 41% on subsequent imaging, ICD (implantable cardioverter defibrillator) evaluation would be readdressed in 3–6 months after goal directed medical therapy. Genetic testing discussions were deferred to outpatient followup as well.

## 3. Discussion

The American Heart Association defines LVNC as a genetic cardiomyopathy [1] while the European Society of Cardiology classifies it as an unclassified cardiomyopathy [4]. This cardiomyopathy may be genetic or sporadically acquired; however, there are still ongoing studies further exploring this.

In the genetic form, LVNC can be an isolated trait or associated with genetic diseases and other congenital defects. Increased risk in family members of affected individuals has been noted from initial studies [5] and genes responsible for some familial cases have been described. Mutations in the* G4.5* gene on the Xq28 chromosome result in a wide range of X-linked cardiomyopathies in the pediatric population such as Barth syndrome and LVNC [6–8]. However, this mutation was not found in the adult population, where autosomal dominance mode of transmission was more common [7–9]. This suggests that presentation of LVNC in adulthood is genetically distinct from pediatric cases. LVNC can be linked to mutations in genes that code for signaling pathway regulators [10], cytoskeletal [11], nuclear membrane, and mitochondrial proteins [12]. Genetic overlap between LVNC and other cardiomyopathies including dilated and hypertrophic cardiomyopathy has been reported as well [7]. These mutations result in a failure of myocytes to compact, resulting in a spongiform appearance with the persistence of deep intertrabecular recesses.

Sporadic forms of LVNC can be acquired later in life. There is emerging data to suggest that increased LV trabeculation fulfilling the criteria for LVNC can be brought on in the setting of increased left ventricular mechanical loading. This can be seen in highly trained athletes [13], pregnancy [14], and sickle cell anemia [15]. Due to limitations in genetic testing, it is unclear if there is a genetic predisposition to the disease in these cases with modifying genes that are able to influence the LVNC phenotype [16]. However, given the common genetic overlap between dilated and hypertrophic cardiomyopathies, which morphologically develop later in life, the hypothesis that this may occur similarly in LVNC should be considered [17].

As noted in our patient, an association between similar facial dysmorphisms of a prominent forehead, low set ears, micrognathia, and high-arching palate and LVNC was first described by Chin et al. [5] in 1990 in a pediatric population. Ichida et al. also describe similar facial findings in a third of the pediatric study population [18]. These patients also had developmental delays as in our patient. Given the strong genetic association in the pediatric population, a link between facial dysmorphisms and development delays in association with LVNC suggests a genetic syndrome, although the chromosome responsible has not been identified.

No associated facial dysmorphisms were seen in two large adult populations with LVNC [2, 3], which can be expected given the studies suggesting that LVNC presenting in adults is genetically distinct from the pediatric population, as described earlier. Our patient's presentation of LVNC in adulthood with associated facial dysmorphisms is unique and rare, suggesting an overlap between the pediatric and adult genetic spectra of LVNC. A possible variable penetrance of the gene mutation or a delayed genetic susceptibility to mechanical loading may also explain this observation, allowing our patient to remain asymptomatic until adulthood.

Our patient was diagnosed initially on echocardiography, which is the first diagnostic tool for LVNC. Several diagnostic criteria have been applied, but the most commonly used one remains a noncompacted/compacted ratio of >2.0 in end-systole [19], although there is no gold standard. Another modality for diagnosis is cardiac magnetic resonance imaging (CMR), which as per the criteria in Petersen et al. [20] uses a ratio of >2.3 in diastole to accurately distinguish nonpathological from pathological noncompaction.

Although left ventricle involvement is much more common, particularly apical, anterior, and inferolateral segments [2, 5], right ventricular (RV) noncompaction has been reported more so in cases where CMR was used in addition to echocardiography for diagnosis [21]. As in our patient, who did not have RV trabeculations on initial echocardiography, the potential role of CMR over echocardiography in evaluation of the RV noncompaction has been suggested before [22] given its enhanced 3-dimensional assessment. Thus, the prevalence of RV involvement may have been underestimated in the past. CMR aids in the diagnosis of biventricular involvement in LVNC although its implications on management are unclear.

There are no specific guidelines or treatment for the management of LVNC. Patients are managed according to their specific clinical need and corresponding guideline. Our patient had all the common clinical complications of LVNC. He presented with heart failure, which is the most common reason for presentation in adults [2, 3]. He was started on an ACE inhibitor and a beta-blocker. His atrial fibrillation, which is common in LVNC, was managed with a rate control strategy prior to spontaneous conversion and he was placed on long-term anticoagulation for his atrial thrombus. The role of anticoagulation is unclear in patients with LVNC and normal LV function. Expert recommendations for primary prophylaxis exist in the setting of arrhythmias, systolic dysfunction [23], or proven atrial or ventricular thrombi as in our patient.

The benefit of ICD placement in this population is unknown as well. The deep recesses of the heart may predispose patients with LVNC to complications, such as ventricular perforation in the setting of device placement. One single study suggests that it is an effective therapy in LVNC for primary and secondary prevention of life-threatening arrhythmias [24]. More studies are needed to determine the safety and efficacy of ICD use in this population. It was considered in our patient but, given his conversion to sinus rhythm without ventricular arrhythmic episodes as well as improvement in EF on subsequent imaging, we felt that a trial of goal directed medical treatment was warranted first. Expert opinions suggest echocardiographic screening of family members as well as genetic testing.

In conclusion, LVNC has a genetic origin that can be found in isolated form or in association with other cardiomyopathies. It can also be acquired later on in life in response to a mechanical stress, perhaps in patients who are genetically predisposed. A better understanding of its genetics and the novel gene mutations implicated may further delineate the natural course of this disease. This will help guide its diagnosis as well as its management. A consensus imaging diagnostic criteria for LVNC are necessary in the context of clinical specifications, given its prevalence in asymptomatic patients [25]. More studies are necessary to determine its optimal management.
